# Design and implementation of a Targeted HealthcaRe InnoVation & Entrepreneurship (THRIVE) fellowship program

**DOI:** 10.1371/journal.pone.0328153

**Published:** 2025-09-24

**Authors:** Ian C. Odland, Joseph Borrello, Layla Fattah, Tyree D. Williams, Kevin D. Costa, David Putrino, Brian Nickerson, Holly Oemke, Turner Baker, James McKay, Dov B. Shamir, Juan Quijano, Janice Gabrilove

**Affiliations:** 1 Mount Sinai BioDesign, Department of Neurosurgery, Icahn School of Medicine at Mount Sinai, Mount Sinai Hospital, New York, United States of America; 2 ConduITS Institute for Clinical and Translational Sciences, Icahn School of Medicine at Mount Sinai, New York, United States of America; 3 Department of Biomedical Engineering, Rensselaer Polytechnic Institute, Troy, New York, United States of America; 4 Cardiovascular Research Institute, Icahn School of Medicine at Mount Sinai, Mount Sinai Hospital, New York, United States of America; 5 Department of Rehabilitation and Human Performance, Icahn School of Medicine at Mount Sinai, Mount Sinai Hospital, New York, United States of America; 6 Department of Population Health Science & Policy, Icahn School of Medicine at Mount Sinai, Mount Sinai Hospital, New York, United States of America; 7 Tisch Cancer Institute, Icahn School of Medicine at Mount Sinai, New York, United States of America; 8 Mount Sinai Innovation Partners, Mount Sinai Hospital, New York, United States of America; NUST: National University of Sciences and Technology, PAKISTAN

## Abstract

The Targeted HealthcaRe InnoVation & Entrepreneurship (THRIVE) Fellowship was created to bridge the gap between healthcare professionals, who often lack experience in technology development and entrepreneurship, and engineers or technology experts, who may not fully understand clinical needs. This eight-month extracurricular program introduces medical and graduate students to the process of health technology innovation. Fellows form multidisciplinary teams to identify and address an unmet clinical need, following Biodesign principles. The program consists of three phases: (1) introduction to healthcare innovation and foundational skills; (2) team formation, mentor selection, and customer discovery; and (3) solution prototyping, pitching, and business plan development. A retrospective analysis of the 2022–2023 cohort evaluated participant demographics, subjective outcomes (Likert-scale surveys on skill acquisition and program satisfaction), and objective metrics (e.g., milestones completed, funds raised, technology disclosures). Descriptive statistics and paired t-tests for pre-post comparisons were used in the analysis. Of the 56 applicants, 29 were accepted, and 20 completed the program. Fellows rated overall satisfaction at 4.45/5, with 85% planning to incorporate healthcare innovation into their future careers. On average, teams met 10.4 of 12 milestones, raised $10,250 in additional funds (in addition to the $5,000 fellowship grant), and filed multiple technology disclosures. Fellows reported significant gains in key innovation skills (p = 1.3E-5) and spent an average of 7.4 hours per week on their projects. The THRIVE Fellowship fosters interdisciplinary collaboration, practical skill development, and a heightened commitment to healthcare innovation. Early successes include strong participant satisfaction, measurable skill acquisition, and substantial external funding. Future program refinements will focus on expanded mentor engagement, enhanced skill-building resources, and long-term tracking of career outcomes. This model may serve as a scalable approach to training future clinicians and researchers in healthcare technology innovation.

## Introduction

Engineering and technology experts aim to grasp the complexities of healthcare delivery, while healthcare professionals often lack significant experience in technology development and entrepreneurship. Furthermore, there are limited opportunities for cross-collaboration between these groups within clinical practice [1]. This gap in communication slows the generation and implementation of practical healthcare technology solutions. One solution is to establish full-time, paid fellowships for post-graduates or industry veterans, such as Stanford’s Biodesign program, while another approach is to offer undergraduate courses or self-limited healthcare hackathons [2–8]. Unfortunately, these opportunities require either dedicated time towards developing these skills, or do not meaningfully integrate trainees into the healthcare technology development ecosystem. A scoping review of innovation programs offered at United States medical schools identified 103 “Innovation and Technology” program across 69 of the 158 accredited allopathic medical schools. Of these 103 programs, only 14 programs were institutes or incubators supporting “interdisciplinary collaboration” [9]. Current systems of medical and graduate training offer limited opportunities for first-time innovators to receive problem-focused cross-disciplinary training. There is a clear need for an extracurricular program that offers an accessible training pathway to develop skills in healthcare technology and entrepreneurship for ambitious, but inexperienced medical and graduate students.

The Targeted HealthcaRe InnoVation & Entrepreneurship (THRIVE) Fellowship is an eight-month program that introduces medical and graduate students from across academic disciplines to health technology innovation. Students form multi-disciplinary teams that work collaboratively to develop a novel technology solution to an unmet healthcare need. The THRIVE Fellowship was piloted at the Icahn School of Medicine at Mount Sinai during the 2020–2021 academic year as a collaboration between ConduITS, Mount Sinai Innovation Partners (MSIP), Icahn School of Medicine Graduate School, and Mount Sinai BioDesign. Conceived as a virtual, longitudinal successor to the Mount Sinai Health Hackathon [6] —a forty-eight-hour team science competition—THRIVE differs from full-time paid fellowships (e.g., Stanford BioDesign, Harvard HealthTech) by allowing students to pursue their primary degree while gaining real-world innovation experience. This approach expands students’ networks across diverse disciplines and institutions, offers longitudinal support beyond traditional hackathons, and reduces extraneous coursework compared to formal university classes. This study aims to assess the outcomes of the 2022–2023 THRIVE cohort, highlighting key successes and challenges. The findings will guide next steps for improvement to ensure ongoing refinement of this accessible, experiential program in healthcare technology innovation.

## Methods

**Fig 1:**
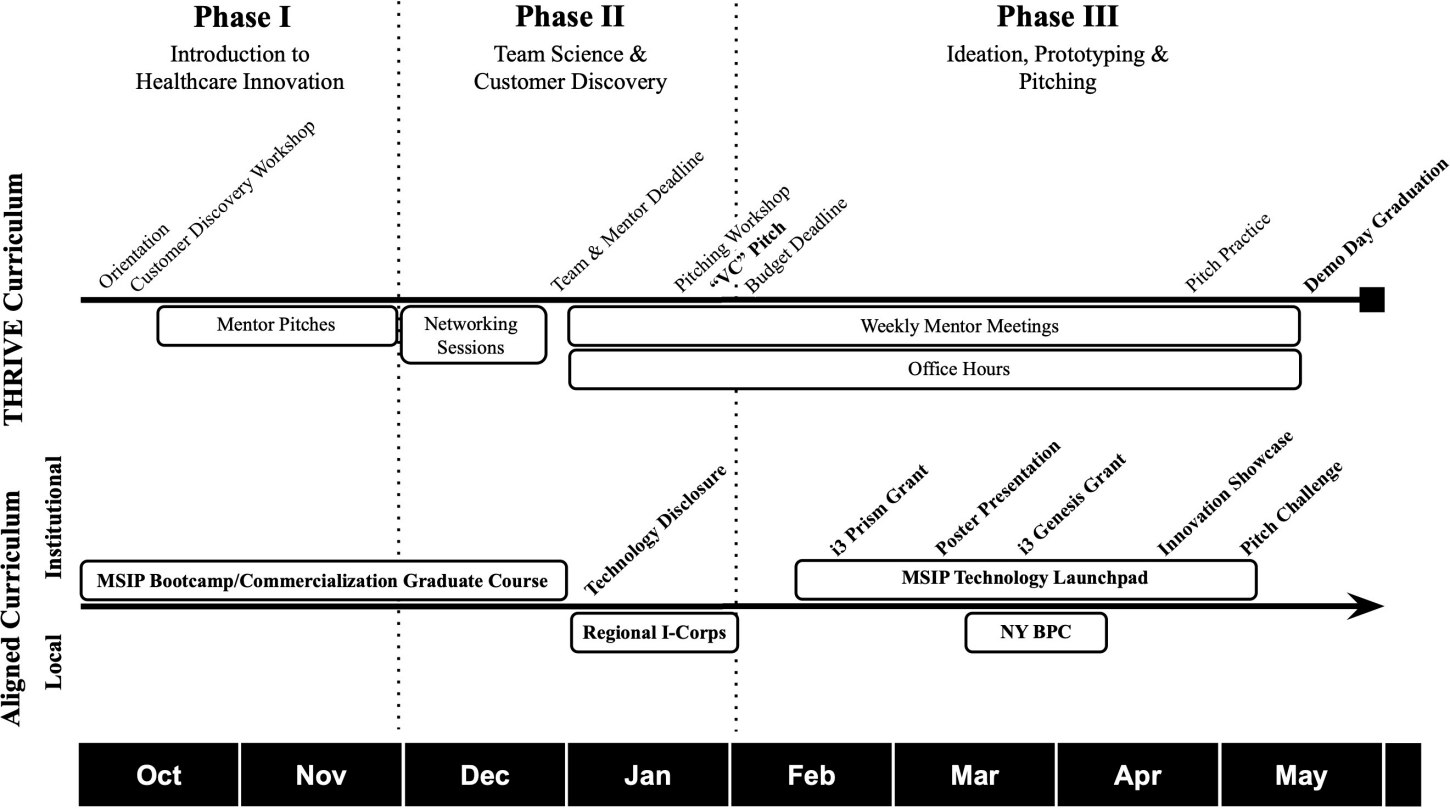
THRIVE Curriculum. Overview of the THRIVE Fellowship and key milestones (**bold**) upon which THRIVE teams were assessed. The first row demonstrates the curricular phases into which the THRIVE curriculum is divided. The second row shows a general overview of the THRIVE curriculum including weekly courses/office hours, deadlines and major program events. The third row highlights the local (above) and institutional (below) programming that was highly encouraged by the THRIVE program.

Inspired by the Biodesign principles described in Yock et al, a three-phase curriculum was created (Fig 1) [1]. The program leverages institutional, local, and national resources such as the MSIP Entrepreneurship Program, a business curriculum offered by the institution’s commercialization office, and National Science Foundation (NSF) I-Corps Regional Course, a three-week customer discovery bootcamp, hosted by the New York Regional Innovation Node (NYRIN) [10–15]. The THRIVE Fellowship aims to develop the next generation of healthcare innovators, while simultaneously developing solutions to unmet clinical needs.

### Phase I: Introduction to healthcare innovation

The first phase (October-November) introduces fellows to peers and faculty mentors, and teaches customer discovery, prototyping and business development skills. An orientation offers fellows an overview of program expectations and resources. A curated lecture series of expert clinicians articulating major clinical needs in their specialty is hosted virtually (Zoom, San Jose, California). Customer discovery training and peer networking sessions are also featured. Fellows are encouraged to build relationships and discuss their ideas asynchronously using Slack (Slack Technologies, San Francisco, California). Fellows may also shadow clinicians to identify healthcare problems and participate in the MSIP Entrepreneurship Bootcamp program, an introductory, extracurricular course on healthcare technology commercialization fundamentals [15–16].

### Phase II: Team science & customer discovery

The second phase (December-January) centers around multidisciplinary team formation, mentor selection, and continued customer discovery. Fellows are instructed to form teams of three to five participants from different professional and academic backgrounds with varying experience and expertise, and partner with a faculty mentor. Teams are instructed to work with their mentor to identify a healthcare-related problem and determine if a sufficient need and market exists for a technology-based solution to that problem. During, or immediately following this phase, teams are highly encouraged to participate in a regional I-Corps program offered by NYRIN. This program requires teams to speak with at least twenty stakeholders to understand the clinical need and potential market and conduct due diligence and competitor analysis. In order to progress to phase III, teams pitch their healthcare-technology idea, project timeline, and budget to the THRIVE leadership committee. Teams are awarded up to $5,000 following a successful project proposal to implement the next stage of their project.

### Phase III: Ideation, prototyping and pitching

The third phase (February-May) focuses on ideation, prototyping, and pitching. Teams attend weekly meetings with THRIVE leadership and complete weekly forms detailing hours worked, goals accomplished, and goals for the following week, in addition to monthly project milestones. Pitch events are hosted to help fellows develop their communication skills and receive formative feedback. Teams are highly encouraged to participate in the MSIP TechLaunchpad program consisting of three pitch competitions with progressively larger monetary prizes: Poster Presentation, Innovation Showcase and Pitch Challenge [15]. Additionally, teams are encouraged to apply for the MSIP i3 Prism and i3 Genesis early research grants and compete in the NYC/NYS Business Plan Competition. During this phase, fellows are also provided access to the Mount Sinai BioDesign rapid prototyping lab [16]. As the final summative assessment, teams must build and present a prototype demonstration for the Fellowship’s graduation demonstration day.

### Mentorship

Fellows benefit from faculty mentors and program mentors. THRIVE teams are required to select a faculty mentor from their home institution, and schedule regular meetings for mentors to provide fellows with clinical/scientific expertise and connections to stakeholders. Faculty mentors support synthesis of customer discovery learning and results from iterative prototyping. Program mentors consist of THRIVE leadership committee members. During Phase III, teams meet weekly with program mentors to provide project updates. Program mentors support participation in extracurricular programming, help fellows resolve interpersonal conflicts, and assist with overcoming challenges and roadblocks. Additional office hours/mentorship are available with business leaders and investors through the MSIP Tech Launchpad to provide students with broader insight into commercializing their solutions.

### Funding and costs

The THRIVE Fellowship is supported by a Clinical and Translational Science Award grant, which allocates an operating budget of $35,000. Up to $5,000 is allocated for prototyping by each team, and unlocked by a successful pitch to the THRIVE leadership committee. The remaining costs are allocated for events and materials shared between all teams.

### Study methods

A retrospective analysis was conducted on the 2022–2023 cohort of the Mount Sinai Targeted HealthcaRe InnoVation & Entrepreneurship (THRIVE) Fellowship. Approval was sought and granted from the Program for the Protection of Human Subjects/ Institutional Review Board at the Icahn School of Medicine at Mount Sinai. All fellows who applied to the program were invited to provide data as part of the application process, and were again invited to provide data through an end-of-program survey administered at the conclusion of the fellowship. Data for the pre-program survey were collected 4–8 weeks prior to the start of official programming, and post-program data were collected 3 weeks following program completion. The total elapsed time between the pre- and post-program surveys was approximately nine months. A retrospective online quantitative survey was used to evaluate the THRIVE Fellowship. The study was determined to be exempt from human research by the Icahn School of Medicine at Mount Sinai Program for the Protection of Human Subjects (IRB Serial Number: 23–00444), and an information sheet was provided to all research subjects.

Kirkpatrick’s framework of training evaluation was used to assess program outcomes, with results reported across four levels: reaction, learning, behavior and results [17]. Survey items included 5-point Likert-scale questions (1 = “strongly disagree” or “very dissatisfied,” 5 = “strongly agree” or “very satisfied”) assessing perceived changes in key innovation-related skills, program satisfaction, and weekly time commitment. Objective metrics, such as number of technology disclosures filed and external funding raised, were also documented.

All statistical analyses were performed in Numbers (Apple, Cupertino, California, USA). Descriptive statistics (mean ± standard deviation) were calculated for both subjective (survey) and objective (milestones) measures. Changes in Likert-scale skill ratings between the start and end of the fellowship were evaluated using paired Student’s t-tests, with α < 0.05 defining statistical significance. Program outcomes were evaluated using Kirkpatrick’s Learning Model, examining participant reaction (satisfaction), learning (skill acquisition), behavior (application of skills), and results (career outcomes and innovation impact).

## Results

### Demographics

*A total of 56* students applied to THRIVE Cohort 3 (2022–2023), of whom 29 (51.8%) were accepted as fellows. In this cohort, 12 (41.4%) self-identified as female and one (3.4%) self-identified as non-binary. Four (13.8%) identified as under-represented in medicine and science. The cohort included 15 (51.7%) were MD students, 6 (20.7%) were PhD students, five (17.2%) were masters students, two (6.9%) were MD/PhD students and one (3.4%) was an employee. The overall dropout rate was 27.4% with 7 (24.1%) exiting the program prior to team formation and one (3.4%) exiting during the team science and customer discovery portion. Four fellows (13.8%) cited limited bandwidth as their reason for dropping out, and four (13.8%) students stopped responding to email and slack correspondence from program leadership. One fellow completed the THRIVE program but was lost-to follow-up for survey at the conclusion of the program. Table 1 shows the demographics of fellows who successfully completed the program compared to those who dropped out.

**Table 1:** Likelihood of fellowship completion according to professional training/background.

	n	Completed	Dropped Out	P-Value
Undergraduate Major				0.41*
Biology/Clinical Medicine	18	12	6	
Engineering/Physics/Math	8	8	0	
Liberal Arts	6	4	2	
Current Degree/program				
MD	15	10	5	0.68**
PhD	7	5	2	
MD/PhD	2	2	0	
MS	5	4	1	
Employees	1	1	0	
Prior career				0.67
Previous career	11	9	2	
No previous career	18	12	6	
Innovation experience				0.67
Previous experience	10	8	2	
No previous experience	19	13	6	
Completed post-graduate degree				1.00
Previous career	12	9	3	
No previous career	17	12	5	

The four processes and nine skills that were tracked among fellows from beginning to end of the THRIVE Fellowship program. *Compared Biology/Clinical Medicine degree to all other degrees combined **Compared MD to all other degrees combined.

### Reaction

Reaction was assessed via an end-of-program satisfaction survey capturing program experience. The survey gathered quantitative data through 5-point Likert scale responses. Fellows rated their overall program experience at 4.45. Fellows rated their experience with faculty mentors and program mentors at 3.7 and 4.7, respectively. Fellows scored the THRIVE Fellowship’s impact on their overall educational experience at 4.55. Additionally, fellows indicated the likelihood of recommending THRIVE to a colleague at 4.58. Fellows rated their overall experience of the program’s education on customer discovery, business model development, medical technology prototyping, pitching ideas and team science as 4.6, 4.0, 4.0, 4.4 and 4.3, respectively.

### Learning

**Fig 2:**
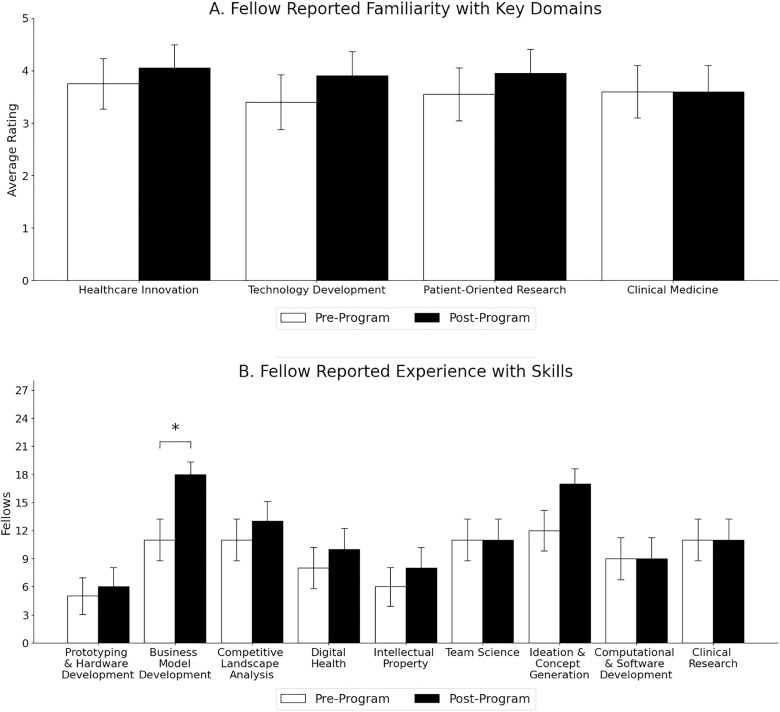
Skills and Experience gained over course of THRIVE Fellowship. Fellow-reported familiarity with tested processes (A) and experience with tested skills (B). Asterisk (*) demonstrate significance at the p ≤ 0.05.

Learning was determined using a pre-post knowledge survey that captured data on exposure to four processes and nine skills (Table 2). This survey gathered quantitative data through 5-point Likert scale responses. Familiarity with healthcare innovation increased from 3.8 to 4.1 (p = 0.21). Familiarity with technology development increased from 3.4 to 3.9 (p = 0.06). Familiarity with patient-oriented research increased from 3.6 to 4.0 (p = 0.18). Familiarity with clinical medicine remained at 3.6 (p = 1.00). In a binary response questionnaire, THRIVE Fellows entered the program with experience in 4.2 skills and exited with experience in 5.2 skills (p = 1.3E-5). Fellow-reported experience with skills slightly increased across most tested aspects, only business model development was statistically significant(p = 0.01)(Fig 2).

**Table 2:** THRIVE Processes and Skills.

Processes
Healthcare Innovation
Technology Development
Patient-Oriented Research
Clinical Medicine
**Skills**
Prototyping & Hardware Development (e.g., Engineering, CAD modeling & 3D printing
Business Model Development/Customer Discovery
Competitive Landscape Analysis
Digital Health (e.g., App Development, Bioinformatics, Data Science)
Intellectual Property
Team Science
Ideation & Concept Generation
Computational & Software Development
Clinical Research

The four processes and nine skills that were tracked among fellows from beginning to end of the THRIVE Fellowship program.

### Behavior

**Fig 3:**
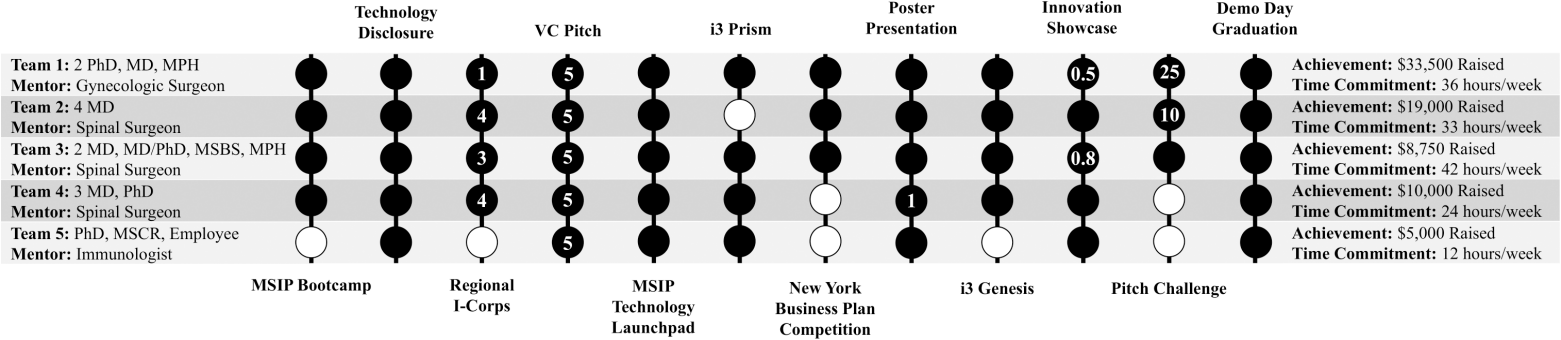
THRIVE team experience over course of cohort 3. Composition, mentorship, milestone achievement, fundraise and time commitment per THRIVE team. Black circles indicate that a milestone was met, white circles indicate that a milestone was not met. The numbers in individual circles indicate the amount of dollars raised through participation in that milestone (in thousands). Please note that teams raised money outside of tracked milestones.

Behavior was gauged based on successful completion of program milestones, which reflected the application of knowledge to practice, along with participants’ intent to participate in healthcare innovation beyond the THRIVE Fellowship. Overall, five teams, ranging from three to five members per team, were formed with three mentors—a spine surgeon, an immunologist and a gynecological surgeon. THRIVE Fellows reported 1.65 team meetings per week. THRIVE Fellows reported working 7.4 (SD:4.0) hours per week on their projects, or 29.4 (SD:11.7) hours per week per team. THRIVE teams accomplished 10.4 (SD:2.1) out of 12 milestones (Fig 3). 85% of fellows agreed that healthcare innovation will be a part of their future careers, and 15% responded that it might be. 75% of fellows stated “that [their] team will continue [their] project following the formal conclusion of the THRIVE fellowship”, with 15% responding “maybe”.

### Results

Results were evaluated based on the funding secured by each team. THRIVE teams raised $10,250 (SD:11,424) per team in addition to the $5,000 awarded to each THRIVE team through the fellowship grant. Fig 3 shows fundraising sources, competition wins, and technology disclosures for each team. This represents $2 raised for every $1 invested. Finally, fellows scored the likelihood that their project will successfully impact patient care at 4 on a 5 point Likert scale.

## Discussion

Our results illustrate the THRIVE Fellowship’s ability to foster interdisciplinary team science and promote successful student integration into the healthcare innovation ecosystem. THRIVE Fellowship is a replicable model that tightly aligns with the National Innovation Network— a NSF initiative of 140 + universities, which the Icahn School of Medicine has been an affiliate site since 2022, across 13 nodes– offering a clear path towards federal grant funding for customer discovery and technical development [11–14]. THRIVE also leverages resources offered by our institution’s commercialization office to enable supplemental programming, award-based pitch competitions and streamlined intellectual property generation.

Feedback indicated overwhelming satisfaction with the curriculum, and behavior demonstrated strong commitment to healthcare technology. The average fellow reported working 7.4 hours/week totaling 6,000 work hours across all fellows. Collectively, teams raised $75,120, indicating significant success pitching their innovation idea to a wider audience. Finally, the program achieved its objective of empowering students to develop a passion for health technology. 85% of graduated fellows indicated that health technology will be a component of their future career, and 75% reported that they will continue working on their project after THRIVE graduation.

**Fig 4:**
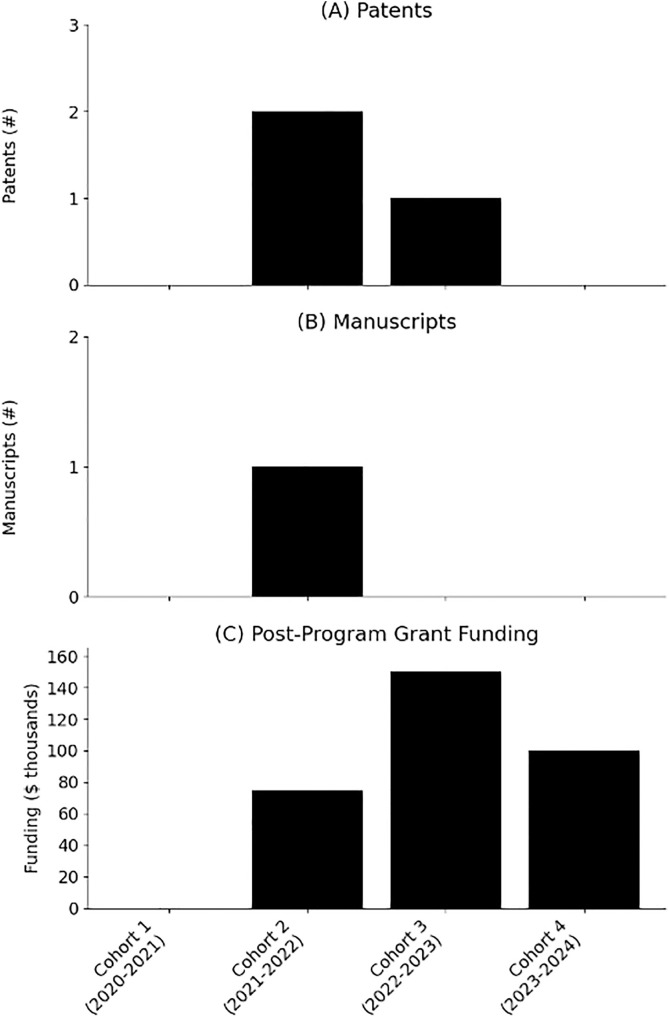
Long-term outcomes from first four THRIVE cohorts including (a) patents, (b) manuscripts and (c) post-THRIVE grant funding.

The key components to program success, in our opinion, is a multidisciplinary student body, an engaged clinical workforce and ideally a rapid prototyping studio, lab or workshop. This has been demonstrated in other programs at the University of Louisville and University of Southern California [18–19]. The Keck Translational Biotechnology Association and the West Coast Consortium for Technology & Innovation in Pediatrics embedded medical students into companies currently developing pediatric medical technologies [18]. Bluegrass Biodesign paired engineering students and medical students to identify clinical problems and brainstorm solutions [19]. Each program generally showed positive program reception and continued interest in technology innovation; but did not show students engaging beyond the program. Importantly, the THRIVE fellowship integrates students into a complete innovation ecosystem that offers funding to support continued product development beyond the confines of the fellowship. Our first four THRIVE cohorts (2020–2024) have resulted in three patent submissions (two international PCT and one provisional applications), $325,000 in non-dilutive grant funding (including $300,000 from six national NSF I-Corps grants), one peer-reviewed manuscript and two technologies in advanced licensing conversations (Fig 4) [20]. Importantly, most academic institutions already offer access to NSF I-Corps programming at both the local and national levels and maintain a technology transfer office to support commercialization. Despite being a single site, retrospective cohort; we believe that our study results can be reasonably generalized to other institutions that share these essential ecosystem components. However, in settings lacking clinical collaboration or hands-on prototyping capabilities, the fellowship’s impact may be attenuated. Thus, while the fellowship model is broadly adaptable, its effectiveness is contingent on institutional readiness and collaboration.

Future iterations of the THRIVE Fellowship will retain the same structure with a few key fellow-recommended changes including more faculty mentor pitches, and strengthened faculty mentor expectations. One challenge we encountered was that three teams selected the same mentor, a returning participant from the previous year. While all teams successfully completed the program, this highlighted the need to expand our mentor pool and better support mentors who are newer to the innovation ecosystem. Moving forward, we plan to feature a broader range of mentors with diverse specialties to better align with student interests and reduce instances of disengagement without follow-up. We also believe that featuring a broader array of clinical specialties will help retain more fellows, particularly medical students, by better aligning with their interests and career goals. Finally, the area with the lowest pre- and post-program self-assessments among THRIVE fellows was prototyping and hardware development. The skills curriculum will be enhanced to help fellows develop additional technical proficiencies. We are currently developing a weekly two-hour long semester extracurricular course that teaches the fundamentals of python coding, computer-aided design, 3D printing and circuit building. We also plan to increase outreach to PhD programs in an effort to attract more students with engineering backgrounds and strengthen the technical skill base of future cohorts.

A major study limitation is that a large portion of the data is self-reported. Applicants may have exaggerated prior experience on the application form used to determine baseline experience and skill. Seven fellows reported a decrease in familiarity with a process and nine fellows reported at least one fewer skill in the exit survey. Another possibility is that as applicants learned more about a space, they reported less experience in accordance with the Dunning-Kruger effect [21]. Importantly, self-reported outcome scores are susceptible to bias. We tried to mitigate this as much as possible by sending our surveys following program completion, and using external programming completion and fundraising as objective outcomes. Additionally, our study has a small sample size (n = 20). There is no long-term data on THRIVE trainee outcomes, however, future studies will track fellows career development.

## Supporting information

S1 Full THRIVE Fellowship Cohort 3 dataset. (XLSX)
