# Correction: Design and implementation of a Targeted HealthcaRe InnoVation & Entrepreneurship (THRIVE) fellowship program

**DOI:** 10.1371/journal.pone.0352774

**Published:** 2026-06-26

**Authors:** Ian C. Odland, Joseph Borrello, Layla Fattah, Tyree D. Williams, Kevin D. Costa, David Putrino, Brian Nickerson, Holly Oemke, Turner Baker, James McKay, Dov B. Shamir, Juan Quijano, Joshua B. Bederson, Janice Gabrilove

Joshua B. Bederson is not included in the author byline. Joshua B. Bederson should be listed as the 13^th^ author and affiliated with Mount Sinai BioDesign, Department of Neurosurgery, Icahn School of Medicine at Mount Sinai, Mount Sinai Hospital, New York, United States of America. The contributions of this author are as follows: Conceptualized research goals and aims, Acquired the financial support for the project leading to this publication, Maintained management and coordination responsibility for the research activity planning and execution, Contributed reagents/materials/analysis tools and Supervised oversight and leadership responsibility for the research.

In Fig 4, the bar graph of Fig 4C is incorrect. Please see the correct Fig 4 here.

**Fig 4:**
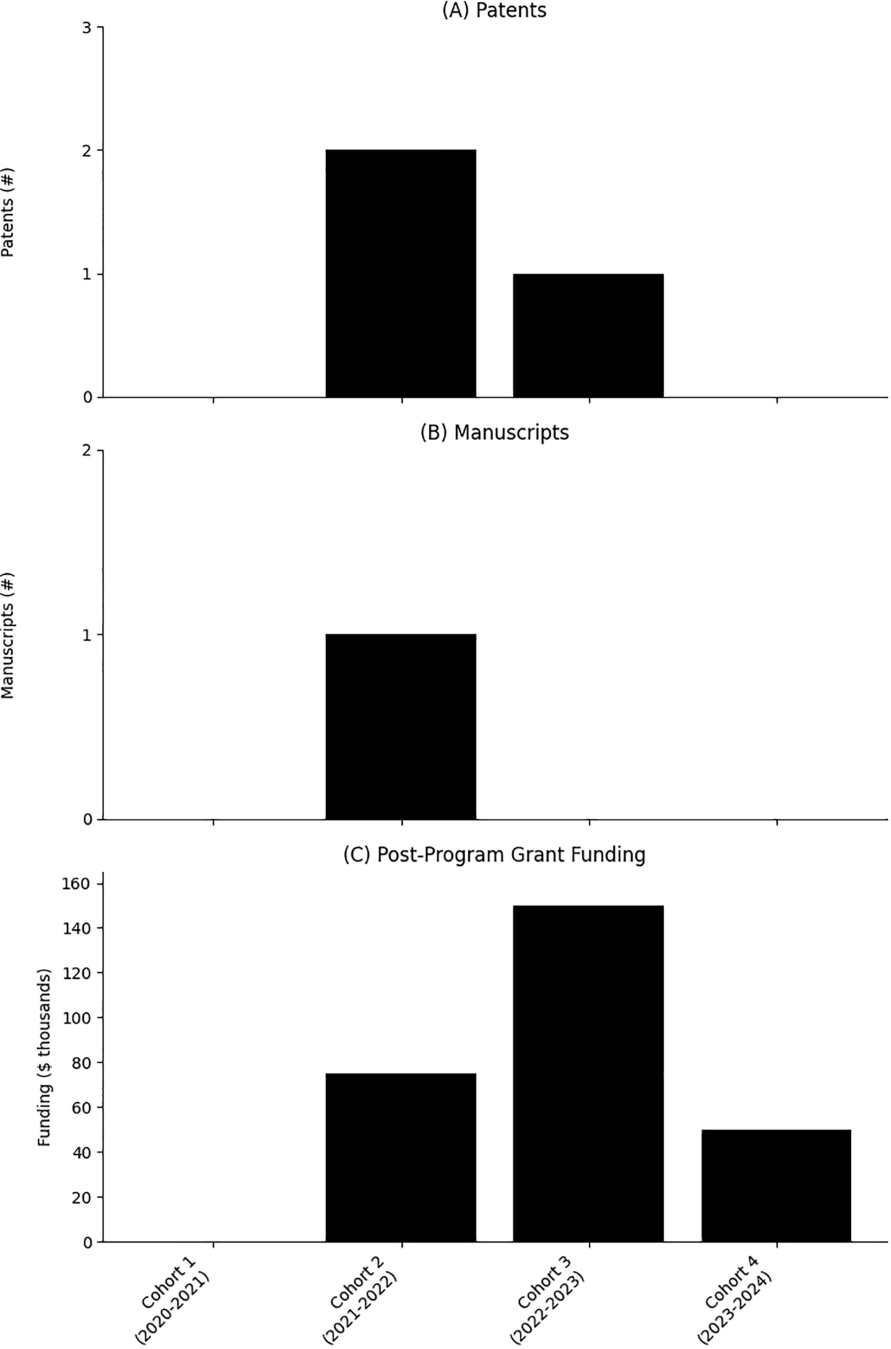
Long-term outcomes from first four THRIVE cohorts including (a) patents, (b) manuscripts and (c) post-THRIVE grant funding.
